# Prognostic factors and survival in parathyroid carcinoma: a 50-year single-center cohort study

**DOI:** 10.20945/2359-4292-2026-0080

**Published:** 2026-07-22

**Authors:** Luiza Helena Crispim da Silva, Letícia Almeida Pontes, Marília D’Elboux Guimarães Brescia, Regina Matsunaga Martin, Felipe Lourenço Ledesma, Hudson Sá Sodré, Venâncio Avancini Ferreira Alves, Fábio Luiz de Menezes Montenegro, Felipe Ferraz Magnabosco

**Affiliations:** 1 Divisão de Cirurgia de Cabeça e Pescoço, Hospital das Clínicas da Faculdade de Medicina da Universidade de São Paulo, São Paulo, SP, Brasil; 2 Divisão de Cirurgia de Cabeça e Pescoço, Unidade de Paratireoide, LIM-28, Hospital das Clínicas da Faculdade de Medicina da Universidade de São Paulo, São Paulo, SP, Brasil; 3 Divisão de Endocrinologia, Hospital das Clínicas da Faculdade de Medicina da Universidade de São Paulo, São Paulo, SP, Brasil; 4 Departamento de Patologia, Hospital das Clínicas da Faculdade de Medicina da Universidade de São Paulo, São Paulo, SP, Brasil; 5 Divisão de Cirurgia de Cabeça e Pescoço, Unidade de Paratireoide, Hospital das Clínicas da Faculdade de Medicina da Universidade de São Paulo, São Paulo, SP, Brasil

**Keywords:** Parathyroid carcinoma, survival rate, prognostic factors, disease-free survival

## Abstract

**Objective:**

This study aims to analyze the clinical characteristics and prognostic factors of patients with parathyroid carcinoma (PC) treated at a tertiary institution.

**Subjects and methods:**

This retrospective cohort included 30 patients with PC who were treated between 1970 and 2023 at a single reference center. Clinical, pathological, and survival data were reviewed and prognostic factors were evaluated.

**Results:**

Of the 30 cases, 16 (53.3%) were women. Mean age at diagnosis was 45.9 years, median follow-up was 60 months and mean tumor size was 33 mm. Median follow-up was 60 months. Disease recurrence occurred in 16 (53.3%) cases, with disease persistence in three (10.0%). Median disease-free survival was 80 months, with a 57% overall survival rate. Neither gender nor age showed significant impact on survival. Patients with lower-stage tumors (T1 by the AJCC/UICC and T1+T2 by the Shaha classification) had significantly better overall survival (p = 0.004 and p = 0.0018, respectively) and disease-free survival (p = 0.03 and p = 0.04, respectively) than those with more advanced stages (p = 0.03 and p = 0.04, respectively). Additionally, patients who experienced tumor recurrence after 36 months had significantly better survival outcomes than those with earlier recurrences (p = 0.02).

**Conclusion:**

Advanced tumor stage (T3 by the Shaha or T2 and above by the AJCC/UICC) is associated with higher mortality in patients with PC. Furthermore, a disease-free interval exceeding 36 months emerged as a relevant factor associated with improved overall survival.

## INTRODUCTION

Parathyroid carcinoma (PC) is a rare malignancy, accounting for less than 1% of primary hyperparathyroidism cases, with fiveand 10-year mortality rates that may reach from about 80% to 50%, respectively (^1^,^2^). It continues to significantly challenge its diagnosis, treatment, classification, and staging.

Differentiating between benign and malignant parathyroid disease can often be difficult from a clinical, radiological, and even histological perspective due to the considerable overlap between these neoplasms. As a result, a definitive diagnosis is frequently established only postoperatively or in case of pathological evidence of metastatic disease. Nevertheless, suspicion of PC can be raised preoperatively based on specific clinical and laboratory findings (^3^). This distinction is critical as the management of these conditions differs significantly, and preoperative biopsy is generally contraindicated due to the risk of tumor seeding, except in cases with documented metastatic disease. Therefore, in cases with suspected PC, *en bloc* resection (removal of the parathyroid gland along with the ipsilateral thyroid lobe and central neck compartment dissection) or, at minimum, resection with negative margins is recommended (^4^).

Several diagnostic classifications for PC have been proposed over the years. In 1973, Schantz and Castleman described a set of histological criteria for PC diagnosis, including capsular or vascular invasion, the presence of fibrous bands, mitotic figures, and a trabecular growth pattern (^5^). Currently, the diagnostic gold standard relies on histopathological features such as vascular, lymphatic, and/or perineural invasion, local invasion into adjacent structures, or metastasis (^6^).

The limited data available regarding the biological behavior and prognosis of PC has hindered the production of an universally accepted staging system that reliably predicts disease-specific survival. However, with the prospective recording and evaluation of new prognostic variables, a formal staging system may be developed in the near future (^7^).

Thus, the rarity of this disease continues to limit the definition of its diagnostic criteria and prognostic factors, which remain under construction via retrospective analyses. Reporting new case series and their clinical outcomes is crucial for advancing this knowledge.

This study aims to find potential prognostic factors - including clinical, laboratory, and histopathological characteristics - associated with PC in a single academic referral center dedicated to managing this disease.

## SUBJECTS AND METHODS

This retrospective cohort study was carried out at *Hospital das Clínicas* at *Faculdade de Medicina da Universidade de São Paulo* (HCFMUSP) - an university public hospital and a reference center for parathyroid disease - with patients who were treated for PC between January 1970 and April 2023.

Demographic data, histopathological features from postoperative pathological analysis, timing, type and outcome of treatment, and use of complementary or adjuvant therapies were evaluated. The following laboratory parameters were analyzed: total serum calcium (CaT) (reference range: 8.4-10.2 mg/dL), ionized calcium (4.49-5.29 mg/dL), and parathyroid hormone (PTH) (15-68.3 pg/mL).

Inclusion criteria comprised patients who had clinical follow-up and/or underwent surgical procedures at the institution during the study period whether as initial treatment or for recurrence. Only patients whose main treatment modality was surgery were included, even if they underwent adjuvant or complementary treatments (such as chemotherapy, radiotherapy, immunotherapy, and/or image-guided interventional ablation), regardless of clinical and/or pathological stage.

In cases in which malignancy was suspected prior to the initial procedure, *en bloc* resection was the recommended surgical approach. This was defined as parathyroidectomy combined with ipsilateral thyroid lobectomy and ipsilateral central neck dissection, preferably performed as a single, uninterrupted specimen.

The diagnosis of PC was based on one or more of the following histopathological criteria: invasion of adjacent structures, unequivocal vascular invasion, presence of metastases, or a previous histopathological diagnosis of PC with recurrence. Atypical tumors or cases with inconclusive findings were excluded from this cohort. All pathology reports were re-evaluated during this study to standardize pathological staging, using the eighth edition of the TNM classification system from the American Joint Committee on Cancer (AJCC) and the Union for International Cancer Control (UICC) (^7^), in addition to the system proposed by Shaha. (^8^) The TNM classification is defined as follows: the primary tumor (T) may be unassessable (Tx) or absent (T0); Tis denotes an atypical parathyroid neoplasm of uncertain malignant potential; T1 tumors are confined to the parathyroid gland with limited soft tissue extension; T2 indicates invasion into the thyroid; T3 corresponds to invasion into the recurrent laryngeal nerve, trachea, esophagus, skeletal muscle, thymus, or adjacent lymph nodes; and T4 represents invasion into major blood vessels or the spine. Regarding regional lymph nodes (N), Nx indicates unassessable nodes; N0, the absence of regional lymph node metastasis; and N1, the presence of such metastases, subdivided into N1a (central or superior mediastinal nodes) and N1b (lateral cervical or retropharyngeal nodes). For distant metastasis (M), M0 denotes absence and M1, the presence of distant spread. Currently, there are insufficient data to establish an anatomical or prognostic staging system for parathyroid carcinoma. On the other hand, According to Shaha’s classification, the staging system for parathyroid carcinoma is defined as follows: T-Tx: not defined by the author; T1: primary tumor <3 cm; T2: primary tumor >3 cm; T3: tumor of any size with invasion of adjacent soft tissues (such as the thyroid or striated muscle); and T4: extensive central compartment disease invading the trachea or esophagus, or recurrent parathyroid carcinoma. N-Nx: not defined by the author; N0: no regional lymph node metastasis; and N1: presence of regional lymph node metastasis. M-Mx: not defined by the author; M0: no evidence of distant metastasis; and M1: evidence of distant metastasis. Based on these parameters, Stage I corresponds to T1N0M0, Stage II to T2N0M0, Stage IIIa to T3N0M0, Stage IIIb to T4N0M0, Stage IIIc to any T, N1M0, and Stage IV to any T, any N, M1.

For patients who underwent initial surgery at external institutions, a paraffin block review was performed by the same pathology team at HCFMUSP whenever possible to maximize the standardization of histopathological assessment. In cases in which this was unfeasible due to unavailable material, specimens from subsequent surgeries were evaluated by the same team to confirm the diagnosis of PC.

Additional variables included age at diagnosis, symptom duration at diagnosis, tumor size and volume, serum calcium and PTH levels at diagnosis and during follow-up, total follow-up time, and time to any recurrence or persistence of disease.

The data were collected and treated on REDCap (Research Electronic Data Capture), hosted at HCFMUSP (^9^,^10^).

Statistical analyses were performed on GraphPad Prism, version 10.5 (GraphPad Software, San Diego, CA, USA). The distribution of continuous numerical variables was tested by the Kolmogorov-Smirnov test. Variables with normal distribution are shown as means with minimum and maximum values or standard deviations and compared using Student’s *t*-test or ANOVA. Non-normally distributed variables are shown as medians with first (Q1) and third (Q3) interquartile ranges and analyzed by the Mann-Whitney or Kruskal-Wallis tests, as appropriate. Categorical variables are shown as frequencies and compared by the chi-squared or Fisher’s exact tests, when applicable. Survival analysis was performed using Kaplan-Meier curves and compared by the log-rank (Mantel-Cox) test. A significance level of 5% was adopted for all p-values.

This study was approved by the Institutional Review Board (Research Ethics Committee of HCFMUSP) and registered at the National Ethics Committee on Human Research under protocol number CAAE 29266720.9.0000.0068.

## RESULTS

The study sample consisted of 30 cases. **Table 1** summarizes their characteristics.

**Table 1:** Patients’ demographic and clinical data

Sex		
Male	14	46.7%
Female	16	53.3%
Age at diagnosis	Mean = 45.9 years	Min-Max = 17-64 years
Time from symptoms to diagnosis	Mean = 24.7 months	Min-Max = 1-96 months
Size (largest diameter) of the tumor	Mean = 36 mm	Min-Max = 15-77 mm
Tumor volume	Median = 6.5 cm^3^	Q1-Q3 = 3.3-8.0 cm^3^
Time to recurrence (locoregional and distant)	Median = 12 months	Q1-Q3 = 8.5-54.0 months
Time to locoregional recurrence	Median = 12 months	Q1-Q3 = 7-36 months
Follow-up time	Median = 60 months	Q1-Q3 = 15.0-95.5 months

This study observed no statistically significant differences when comparing sex with overall survival (Log-rank, p-value = 0.55) or disease-free survival (Log-rank, p-value = 0.74).

This research analyzed patients in two groups: those younger and those older than 45 years of age (a cut-off chosen based on the mean age of the cohort). Serum calcium, PTH levels, and time to recurrence showed no statistically significant differences between these groups. Although younger patients had smaller tumors, this failed to impact survival outcomes. This research observed no statistically significant difference between these age groups regarding disease-specific mortality versus being alive or deceased from other causes (Fisher’s exact test, p-value = 0.69). Similarly, it found no significant difference for these outcomes between genders (Fisher’s exact test, p-value = 0.69). The comparison of disease-free survival between age groups found no relevant associations (Log-rank, p-value = 0.66).

**Figure 1:**
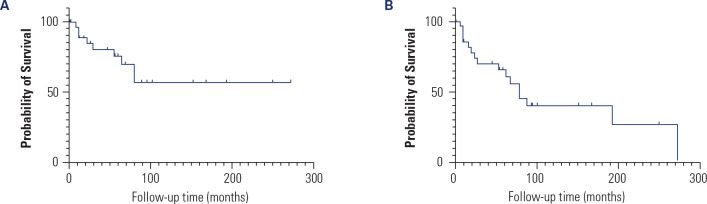
Kaplan-Meier curve for (**A**) overall survival and (**B**) disease-free survival in patients diagnosed with parathyroid carcinoma.

The median disease-free survival totaled 80 months, after which the overall survival rate equaled 57% (**Figure 1**).

The mean total CaT and PTH levels (with ranges) totaled 14.5 mg/dL (11-19 mg/dL) and 1,470 pg/mL (158-3,654 pg/mL), respectively. Higher serum calcium levels showed no association with statistically significant impact on survival. In the gender-based analysis, no assessed variable reached statistical significance, although CaT levels tended to be slightly higher among men.

This study found no correlation between tumor size (greater or smaller than 33 mm) and overall (Log-rank, p-value = 0.96) or disease-free survival (Log-rank, p-value = 0.57).

Regarding mortality (death from disease vs. not deceased from disease by the end of follow-up), this study found no correlation with vascular invasion (Fisher’s exact test, p-value = 0.55). Similarly, we observed no statistically significant correlation between vascular invasion and overall (Log-rank, p-value = 0.21) or disease-free survival (Log-rank, p-value = 0.34), despite an apparent trend.

**Figure 2:**
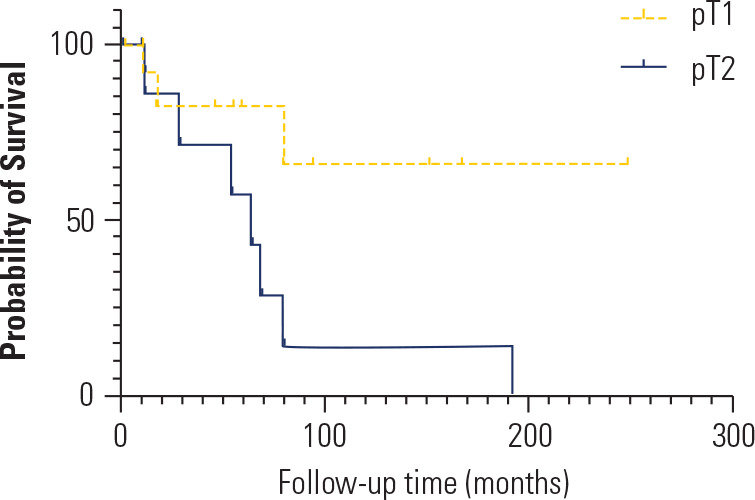
Kaplan-Meier curve for disease-free survival among patients with T1 and T2 stages in the AJCC/UICC classification.

For AJCC/UICC staging, **Table 2** summarizes the distribution by T and N stages. Regarding distant metastasis, 28 patients (93.3%) had no evidence of distant metastases (M0), whereas only two (6.7%) showed systemic disease (M1). Statistical analysis compared patients with T1 and T2 tumors, excluding T3 cases and those with distant metastasis at diagnosis due to the small sample size for proper statistical inference. This comparison found a significant difference for mortality (Fisher’s exact test, p-value = 0.004) and disease-free survival (Log-rank, p-value = 0.03) (**Figure 2**).

**Table 2:** Distribution of cases by T and N staging according to the AJCC/UICC and the classification proposed by Shaha

	AJCC/UICC	Shaha
Initial T		
T1	15 (50.0%)	9 (34.6%)
T2	8 (26.6%)	5 (19.2%)
T3	3 (10.0%)	9 (34.6%)
T4	0 (0.0%)	2 (7.7%)
Tx	4 (13.3%)	Not defined by the authors
Initial N		
N0	29 (93.3%)	25 (96.1%)
N1	1 (3.3%)	1 (3.8%)
Nx	0 (0.0%)	Not defined by the authors
Staging		
I	Not defined by the authors	9 (34.6%)
II	Not defined by the authors	5 (19.2%)
IIIa/b	Not defined by the authors	10 (38.5%)
IV	Not defined by the authors	2 (7.7%)

**Figure 3:**
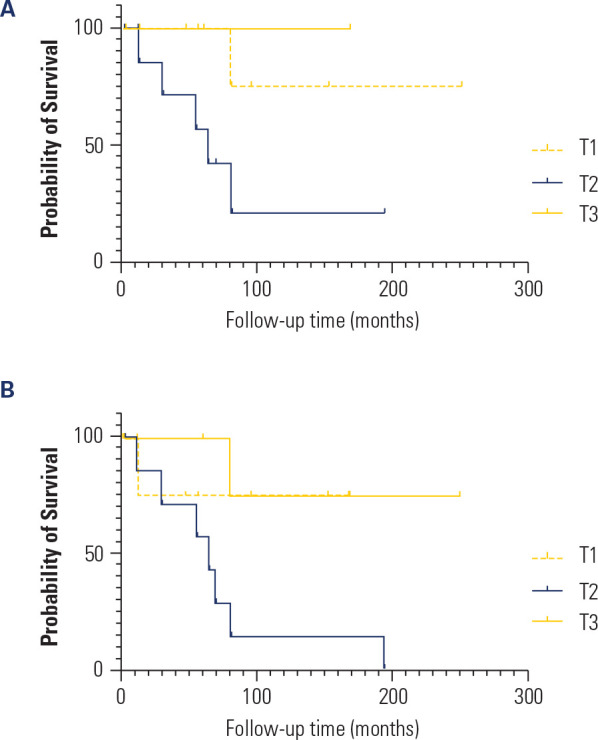
Kaplan-Meier curves for (**A**) overall survival and (**B**) disease-free survival among patients with T1, T2, and T3 stages in the Shaha classification.

This study also evaluated patients according to the Shaha staging system (**Table 2**). It excluded four patients due to insufficient diagnostic information for accurate staging classification (such as maximum tumor size). This staging model, grouping T1 and T2 together (due to sample size) versus T3, observed a statistically significant difference between the groups, with higher mortality (Fisher’s exact test, p-value = 0.0018) and shorter disease-free survival (Log-rank, p-value = 0.04) in the latter group (**Figure 3**).

**Figure 4:**
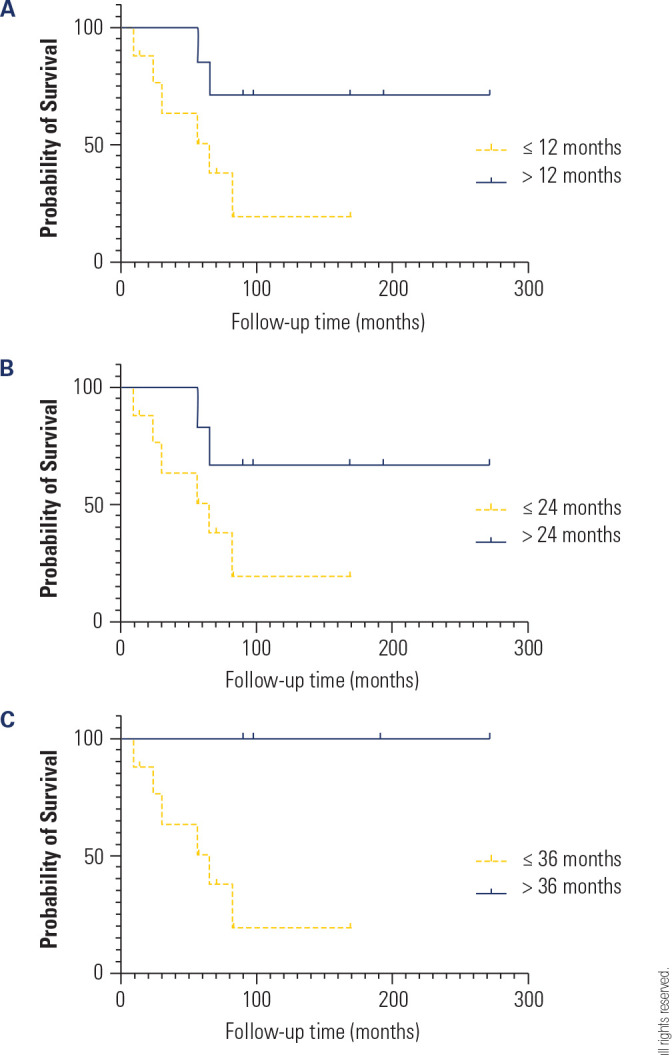
Kaplan-Meier curves for overall survival of patients who showed recurrence during follow-up according to the time separation point at which they occurred: (**A**) 12, (**B**) 24, and (**C**) 36 months.

Finally, this study analyzed the impact of time to recurrence on survival regardless of whether the recurrence was locoregional or distant. Considering only patients who experienced recurrence at any point during follow-up and applying temporal cut-offs at 12 and 24 months, we found no statistically significant difference in mortality between these groups (Log-rank, p-value = 0.06 and 0.10, respectively). However, analysis of the 36-month time point found a statistically significant difference in mortality (Log-rank, p-value = 0.02), indicating lower survival among patients who recurred within this period (**Figure 4**).

## DISCUSSION

Several studies using population-based databases have investigated prognostic factors in patients with PC. Sadler and cols. analyzed a cohort of patients diagnosed with PC over the years and found that the presence of metastatic lymph nodes at diagnosis and age were associated with disease survival (^11^). Similarly, Asare and cols., analyzing the same population-based dataset, observed an association between age, sex, and tumor size with survival in patients with PC (^12^). However, this cohort showed no such associations, probably due to its small sample size.

Ohkuwa and cols., in a recent single-center study with a patient number comparable to ours, found three predictive factors for recurrence and prognosis in patients with PC: Ki-67 index, weight of the affected gland, and presence of necrosis on histopathological examination (^13^). According to the authors, a Ki-67 index ≥4.1%, gland weight ≥4,890 mg, and the presence of necrosis may be associated with distant metastasis and increased recurrence risk.

The literature has no consensus on tumor size as a prognostic factor in PC. Hsu and cols. concluded that a tumor size ≥3 cm is associated with lymph node metastasis (^14^). Additionally, Asare and cols. correlated tumors ≥4 cm with increased mortality risk (^12^). Qian and cols., in a multicenter study using surveillance and epidemiological data from 604 PC patients, confirmed that tumors ≥3.5 cm were associated with worse overall survival (^15^). However, Ohkuwa and cols., in a smaller study, failed to confirm this association (^13^). Similarly, Lee and cols. also found no relationship between tumor size and survival (^16^). The HCFMUSP cohort in this study was also unable to establish such associations.

To address the need for standardized classification and subgrouping of patients based on prognosis (staging), several systems have been proposed over time. Notable among them are those proposed by Shaha and Shah (^8^), Talat and Schulte (^17^,^18^), and more recently, the AJCC/UICC TNM staging system (^7^).

Despite many proposed staging systems, all still require validation before they can be widely adopted. The TNM system (AJCC/UICC), for example - widely used for many cancer types - currently includes no detailed PC subclassifications within stages, mainly due to insufficient data. The Shaha system, on the other hand, differs by incorporating tumor size. The Schulte system stratifies prognosis based on histopathological features, such as vascular invasion. Thus, while all these systems aim to stratify patients prognostically, they emphasize different disease characteristics. Validation and analysis of such systems in various cohorts and for distinct clinical or pathological variables are essential to strengthen existing models and contribute to the overall understanding of the disease.

Within our cohort, comparing T1 and T2 tumors (excluding patients with M1 disease at diagnosis) according to the AJCC/UICC TNM system obtained a significant association with survival, confirming that tumors extending beyond the parathyroid gland are linked to worse survival outcomes.

The analysis using the Shaha system grouped T1 and T2 cases as the limited number of patients and the relatively short follow-up period made statistically significant analysis challenging. Nevertheless, this study observed that patients with early-stage tumors (T1 and T2) had better overall and disease-free survival than those with more advanced tumors (T3) by this classification.

A widely used histopathological parameter to predict prognosis in other head and neck cancers refers to the presence of vascular invasion. Although this is often a decisive prognostic criterion in other tumor sites, this analysis observed no such association. This may be because vascular invasion is a diagnostic criterion for PC, and the qualitative assessment - classified simply as present or absent - without quantification in this study may have limited its prognostic evaluation. This, for example, hindered a more robust assessment of the Schulte system. Perhaps, applying a grading system for vascular invasion (e.g., invasion in ≤5 *foci versus* >5 *foci*) would have found a significant association with disease-free survival.

Another prognostic factor, first highlighted by Schantz and Castleman in 1973 (^5^), is the time to recurrence. However, due to small sample sizes in most series, some authors have been unable to corroborate this finding. Therefore, while clinically relevant, this aspect remains controversial. Despite having been suggested decades ago and representing an important biological marker of PC aggressiveness, the relationship between disease-free interval and mortality has been insufficiently investigated in recent years.

A study reviewing 47 years of institutional data, including 37 cases from 1966 to 2009, found no association between mortality and time to first recurrence. Instead, it associated mortality with the presence of lymph node or distant metastases, higher calcium levels at recurrence, and higher levels of hypocalcemia-inducing medications (^19^). Conversely, a 2022 literature review with studies from 1966 to 2019, including a total of 73 patients, suggested that disease-free interval was indeed a survival predictor (^20^).

Similarly, a previous smaller cohort from the same institution has shown that the time between initial treatment and development of recurrent disease (locoregional or distant) negatively affected overall survival rates (^21^). The difference was marginal and statistically insignificant at 24 months but became clearly significant for recurrences within 36 months, resulting in poor survival (five-year median). This expanded cohort corroborated this finding. The evaluation of survival regarding time to disease recurrence (locoregional or distant) observed no significant (although borderline) relationship with mortality when recurrence occurred before or after 12 or 24 months. However, the analysis of recurrence within 36 months of the initial treatment confirmed a statistically significant association with mortality. This result reinforces the idea that time to recurrence is related to patient survival, especially when recurrence occurs within the first three years of follow-up. The possible explanation is that PC may have different molecular mechanisms and biological behaviors.

Although the occurrence of parathyroid carcinoma is relatively uncommon, HCFMUSP has gained substantial experience in managing these patients over a 50-year period. Despite the challenges inherent to evaluating such a rare condition, the surgical approach and its outcomes, along with the underlying principles, have remained largely consistent from the beginning of the cohort to the present day (^21^,^22^). From the outset, the recommended management for these patients has been *en bloc* resection to maximize the likelihood of cure and effective treatment. Over time, however, greater experience has been achieved in identifying patients with an increased suspicion of malignancy, enabling more oncologically appropriate therapeutic strategies.

The clinical implications of these findings lie in the potential use of staging and recurrence timing as practical tools for risk stratification and patient counseling. By integrating these parameters into clinical decision-making, physicians in referral centers may enhance follow-up strategies and provide more accurate prognostic information to patients with PC.

Despite representing one of the larger single-center PC cohorts in the literature - especially given the rarity of the disease - this study has certain limitations. First, its small sample size limited its ability to perform robust analyses. Although its small sample size represents an inherent limitation due to the rarity of parathyroid carcinoma, these data contribute valuable clinical insights that may support future meta-analyses or multicenter studies aimed at increasing statistical power and refining treatment strategies. The fact that the cohort comes from a single institution and that some initial surgeries were performed elsewhere adds to this limitation. Moreover, due to the general lack of knowledge about the disease, no standardized treatment protocol was consistently applied, with management strategies varying according to the attending team’s judgment over a long observation period. Although inherent to the long study period and the rarity of parathyroid carcinoma, this study mitigated such variability by applying adequate surgical and pathological criteria throughout the cohort. Future efforts should aim to develop standardized treatment protocols or even to set up a national registry to facilitate comparisons across institutions and improve patient outcomes. Finally, the lack of centralized histopathological review - particularly for patients initially treated at external centers - hindered a more uniform pathological assessment (81% of the cases had pathology slides available from either primary or subsequent operations for histopathological re-evaluation).

In conclusion, PC presents with heterogeneous biological behavior, with some patients surviving for decades and an indolent disease course in a significant proportion of cases. It is associated with potentially severe clinical findings and carries a considerable risk of recurrence, including the possibility of late metastases. Additionally, patients diagnosed with T3 (according to the Shaha classification) or T2 and above PC (according to the 2017 AJCC/UICC TNM staging system) have higher mortality than those with earlier stages of the disease. Furthermore, a disease-free interval longer than 36 months proved to be a relevant factor associated with improved overall survival. These findings, derived from one of the largest Brazilian referral centers, add valuable information to the limited global literature on this rare malignancy and underscore the importance of further multicenter and collaborative studies to refine prognostic assessment and management.

## Data Availability

datasets related to this article will be avail-able upon request to the corresponding author.
